# Feline infectious peritonitis: A comprehensive evaluation of clinical manifestations, laboratory diagnosis, and therapeutic approaches

**DOI:** 10.5455/javar.2024.k742

**Published:** 2024-03-12

**Authors:** Wassamon Moyadee, Supita Sunpongsri, Kiattawee Choowongkomon, Sittiruk Roytrakul, Amonpun Rattanasrisomporn, Natthasit Tansakul, Jatuporn Rattanasrisomporn

**Affiliations:** 1 Graduate Program in Animal Health and Biomedical Sciences, Faculty of Veterinary Medicine, Kasetsart University, Bangkok, Thailand; 2 Department of Companion Animal Clinical Sciences, Faculty of Veterinary Medicine, Kasetsart University, Bangkok, Thailand; 3 Kasetsart University Veterinary Teaching Hospital, Faculty of Veterinary Medicine, Kasetsart University, Bangkok, Thailand; 4 Department of Biochemistry, Faculty of Science, Kasetsart University, Bangkok, Thailand; 5 Functional Proteomics Technology Laboratory, Functional Ingredients and Food Innovation Research Group, National Center for Genetic Engineering and Biotechnology, National Science and Technology for Development Agency, Pathum Thani, Thailand; 6 Interdisciplinary of Genetic Engineering and Bioinformatics, Graduate School, Kasetsart University, Bangkok, Thailand; 7 Department of Pharmacology, Faculty of Veterinary Medicine, Kasetsart University, Bangkok, Thailand

**Keywords:** Cats, coronavirus, effusion, feline infectious peritonitis, prednisolone

## Abstract

**Objective:**

This study aimed to investigate the clinical and laboratory characteristics of naturally occurring feline infectious peritonitis (FIP) and estimate the median survival time of FIP cats treated with prednisolone to guide further therapeutic planning.

**Materials and Methods:**

In this retrospective study, data from a total of 116 cats with effusion were fully recorded. Forty-five FIP-diagnosed cats were enrolled for analysis.

**Results:**

The study findings indicate that FIP was a disease affecting cats aged 1–2 years and was highly prevalent among male cats. Clinical manifestations of FIP affected the digestive (60%), hematological (53.3%), respiratory (33.3%), neurological (6.7%), and ocular (4.4%) systems. Blood profiles revealed mild anemia, lymphopenia, thrombocytopenia, hypoalbuminemia, hyperglobulinemia, and an albumin to globulin ratio of 0.4. Fluid analysis and cytology of FIP cats demonstrated a transparent yellow fluid with a protein content of 6 gm/dl and a total nucleated cell count of approximately 5,000–10,000 cells. During the observation period, FIP cats treated with prednisolone exhibited a median survival time of 31 days.

**Conclusion:**

Confirming FIP cases can be challenging; therefore, a tentative diagnosis of FIP must be made with care. This study provided practical diagnostic tools to diagnose FIP based on clinical signs and multiple abnormalities, which allowed for more efficient and rapid detection.

## Introduction

Feline infectious peritonitis (FIP) and COVID-19 are two distinct diseases caused by different coronaviruses and affecting different species [1]. Common features are observed in both FCoV and SARS-CoV-2, such as the rapid spread of infection, the potential to decrease infection rates by isolating infected patients, and the shared efficacy of comparable anti-inflammatory or antiviral compounds [1,2]. A virulent FCoV, or feline infectious peritonitis virus (FIPV), is disseminated via the mononuclear phagocyte system, while a nonvirulent FCoV is associated with asymptomatic persistent enteric infections [3]. Host factors involved in FIP were stressful events, including surgery, rehoming, multi-cat households, and retrovirus co-infection, which may increase the susceptibility of cats to developing FIP [1–3].

Currently, there is no single diagnostic test available that can accurately diagnose every case of FIP, and a definitive diagnosis is still difficult. The literature has presented an algorithm for diagnosing FIP, including the environment, clinical signs, laboratory findings, serological tests, and viral antigen detection [3–5].

Historically, there have been three main generally accepted therapies for FIP, including glucocorticoids or immunosuppressive drugs, nonspecific immunostimulant drugs, and antiviral agents [3,6]. In the past, the treatment aimed to control inflammation and hypersensitivity triggered by FCoV [3,6]. Thus far, the treatments aim to act against coronavirus infection, increase survival rates, and achieve a clinical cure [7]. Despite the lack of specific treatments or commercially available effective antiviral medications such as GS-441524, remdesivir, and molnupiravir, prednisolone has been prescribed as an acceptable treatment for FIP [6,7]. This study is designed to investigate common clinical signs and laboratory features, as well as to evaluate the therapeutic effects of prednisolone on cats with FIP. Additional research and clinical trials are necessary to fully understand the potential benefits and risks associated with the use of prednisolone in managing both COVID-19 and FIP.

## Materials and Methods

### Ethical approval

This study was approved by the Ethics Committee of Kasetsart University (ID# ACKU60-VET-014), and all cat owners provided written consent.

### Animal and study design

The medical data of 116 cats with effusion visiting Kasetsart University Veterinary Teaching Hospital (KUVTH) between December 2016 and October 2022 were retrospectively analyzed. Forty-five highly suspected FIP cases, with randomized age, breed, and sex, along with full medical records, were collected for analysis. Due to limited facilities and the cat owners’ refusal to undergo invasive diagnostic procedures or necropsy examinations, most cases were not confirmed by immunohistochemistry (IHC). The diagnostic algorithm used to confirm FIP included the typical clinical signs of effusive FIP, the presence of pleural or peritoneal effusion, a decreased albumin to globulin (A:G) ratio below the cutoff point (0.8 in serum and effusion), fluid analysis showing inflammatory cells and protein-rich content, a positive Rivalta’s test, and polymerase chain reaction analysis for FCoV antigen detection [8–10]. All cats tested negative for feline leukemia virus (FeLV) and feline immunodeficiency virus (FIV) using the commercial test kit Witness^®^ FeLV/FIV (Zoetis, USA). Signalments, clinical signs, hematology, blood chemistry, fluid analysis, and cytology were thoroughly evaluated. The median survival time was calculated from the first day of FIP diagnosis until the last visit or the death of the cat. The packed cell volume (PCV), total protein, albumin, and globulin levels were compared at two different time points.

### Statistical analysis

All statistical analyses were performed using NCSS software (version 2007, NCSS statistical software, USA). Descriptive statistics were employed for all evaluated variables. *P* < 0.05 was considered statistically significant.

## Results

### Signalments and clinical signs

**Figure 1.**
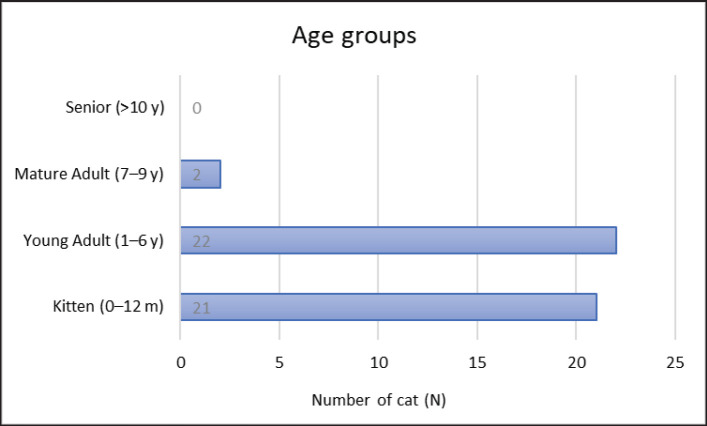
The age group of the strongly suspected FIP cases. The number of cats is presented on the X-axis.

Of the 45 FIP cats, the age of the affected cats ranged from 4 months to 9 years, with an average age of 1.6 years and a median age of 1.1 years. Among these cases, 48.9% were young adults, 46.7% were kittens, and 4.4% were mature adults (Fig. 1). The study found that FIP cats were significantly more likely to be male, with a prevalence of up to 73.3% compared to the overall feline population. Out of the 45 cats, 10 (22.2%) were purebred, including breeds such as Scottish fold (5), Persian (4), and Bengal (1). The remaining 77.8% were domestic shorthair cats.

**Figure 2.**
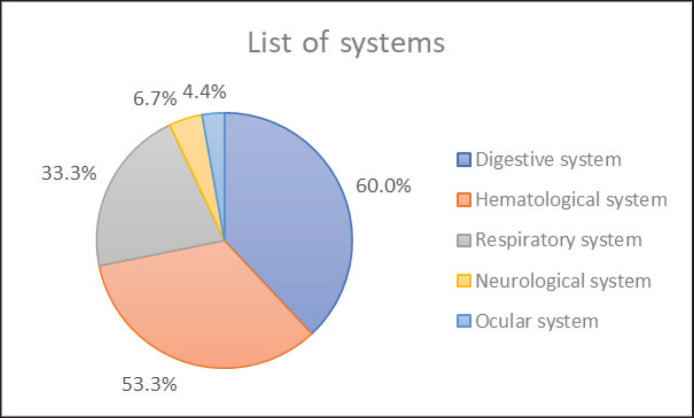
Frequency of clinically affected systems of strongly suspected FIP cats. The digestive system included abdominal distension, diarrhea, and vomiting. The hematological system included pale mucous membrane, anemia, icterus, and dehydration. The respiratory system included dyspnea and panting. The neurological system included paresis, paralysis, ataxia, and seizure. The ocular system included uveitis, corneal edema, anisocoria, and retinal detachment.

The clinical findings obtained in this study, separated by body systems, showed 60% digestive system (abdominal distension, diarrhea, and vomiting), 53.3% hematological system (pallor or anemia, icterus, and dehydration), 33.3% respiratory system (panting and dyspnea), 6.7% neurological system (ataxia, seizure, behavior changes, paralysis, and twitching), and 4.4% ocular system (uveitis, nystagmus, corneal edema, anisocoria, and retinal detachment) (Fig. 2). Peritoneal effusion, pleural effusion, and bilateral fluid accumulation were observed in 28, 14, and 3 cats, respectively. The main clinical signs categorized according to the body systems were abdominal distension, pallor or anemia, dyspnea, behavior changes, and uveitis.

### Hematology and serum chemistry

The mean, median, and range for the complete blood count and serum biochemistry of strongly suspected FIP cats are presented in Table 1. PCV decreased in 73.3% of the cats, hemoglobin (Hb) decreased in 66.7% of the cats, but only 22.2% of the cats had a decrease in red blood cell (RBC) count. The white blood cell (WBC) count increased in 13.6% of cats with FIP. Lymphopenia developed in 75% of cats with FIP, while 45.5% showed neutrophilia, and 13.6% had monocytosis. In all cats with a full hematological evaluation, no blood parasites in the blood film or positive saline autoagglutination were observed.

**Figure 3.**
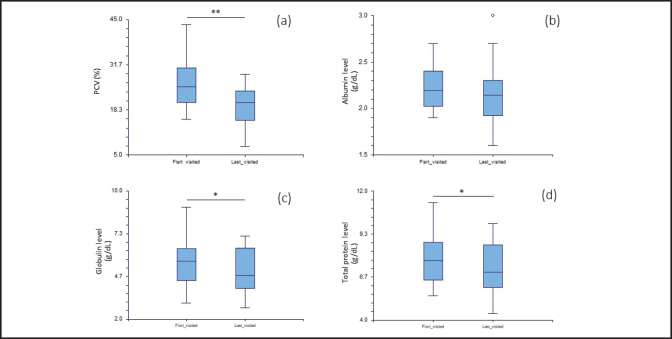
Box plot of (a) PCV, (b) albumin levels, (c) globulin levels, and (d) total protein levels between the initial and final visits of cats diagnosed with effusive FIP. Boxes represent the interquartile range, while the horizontal bar within each box represents the median. Upper and lower bars and outliers (opened circles) are plotted using NCSS software.

In serum biochemistry, 33.3% of the cats showed increased levels of total protein. Hypoalbuminemia was observed in almost all FIP cats (91.1%). Hyperglobulinemia was found in 60% of cats strongly suspected of having FIP. The A:G ratio ranged from 0.25 to 0.7, with a mean of 0.43 and a median of 0.40. Among the results, 37.8% had ratios lower than 0.4%, 51.1% had ratios between 0.4% and 0.6%, and 11.1% had ratios between 0.6 and 0.8. Alanine aminotransferase (ALT) levels were mostly normal (83.3%). Creatinine concentrations decreased in 69% of the cats, while 62.9% had low blood urea nitrogen (BUN) levels. None of the cats showed an increase in BUN. Different variables of FIP cats (PCV, albumin, total protein, and globulin) at the first and last presentations are shown in Figure 3. The values of PCV at the final visit were significantly lower (*p* < 0.01) than at the initial visit (Fig. 3a). The levels of total protein and globulin at the first and last presentations revealed statistical differences between the two-time points (*p* < 0.05). However, no significant difference was found in albumin levels or the A:G ratio between the first and last visits.

### Fluid analysis and cytology

Fluid analysis and cytological examination of the 45 cats revealed the presence of effusion characterized by high protein concentrations and nonspecific inflammatory cells. The effusion is typically yellowish, resembling straw or amber-colored staining. However, the total nucleated cell count (TNCC) and A:G ratio for two of these cats were unavailable. As shown in Table 2, the TNCC ranged from 384–50,100 cells/μl, with a mean of 9,207 cells/μl and a median of 5,230 cells/μl. The effusion contained a mixture of nondegenerated neutrophils, macrophages, and some lymphocytes. Protein in effusion showed high levels at a mean of 6.14 and a median of 6.0, respectively. The effusion A:G ratio varied from 0.22 to 0.7, which was similar to the mean and median ratios observed in the serum.

**Table 1.** Hematology and blood chemistry.

Parameters	Normal reference	Number of animals	Mean	Median	Range
Hb (mg/dl)	9.8–15.4	45	9.3	8.41	4.8–15.1
RBC (×10^6^/µl)	5.0–10.0	45	6.7	6.4	2.8–13.6
PCV (%)	30–45	45	27.2	25.7	16.7–44.3
WBC (×10^3^/µl)	5.5–19.5	45	17.2	13.5	3.8–61.1
Neutrophils (×10^3^ µl)	2.5–12.5	44	14.9	12.1	2.8–53.8
Lymphocytes (×10^3^/µl)	1.5–7	44	1.3	0.9	0.1–5.4
Monocytes (×10^3^/µl)	0–0.9	44	0.4	0.3	0.0–1.6
Eosinophils (×10^3^/µl)	0–0.8	44	0.1	0	0.0–0.7
PLT (×10^3^/µl)	200–800	41	268.1	241.5	101–689
BUN (mg/µl)	19–34	35	18.94	17	11–82
Creatinine (mg/dl)	0.9–2.2	42	0.89	0.85	0.36–1.75
ALT (U/l)	25–97	42	75.19	35	11–475
Total protein (gm/dl)	6–7.9	45	7.7	7.6	4.3–11
Albumin (gm/dl)	2.6–4.2	45	2.1	2.18	1.5–3.1
Globulin (gm/dl)	2.6–5.1	45	5.63	5.3	5.3–9
A:G ratio		45	0.42	0.4	0.2–0.7
<0.4		17 (37.8%)			
≤0.4–0.6		23 (51.1%)			
0.6–0.8		5 (11.1%)			

ALT= alanine aminotransferase, A:G ratio= albumin to globulin ratio, BUN= blood urea nitrogen,

Hb= hemoglobin, PCV= packed cell volume, PLT= platelets. RBC= red blood cells, WBC= white blood cells.

* *p* < 0.05, ** *p* < 0.01 PCV = packed cell volume.

### Treatment and survival time

**Figure 4.**
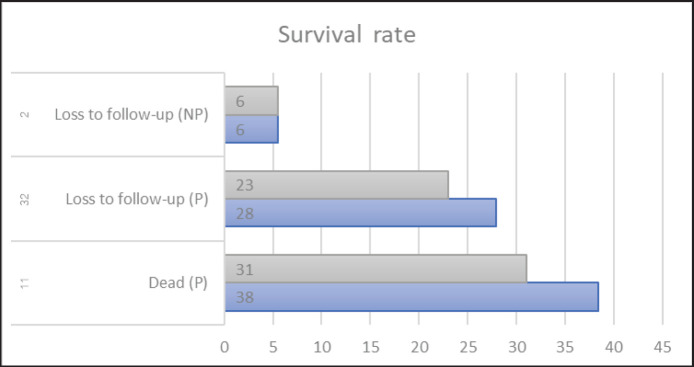
Mean and median survival time of FIP cats treated with prednisolone (P) at an immunosuppressive dosage of 2 mg/kg/day, compared to cats receiving only symptomatic treatments (no prednisolone, NP).

As shown in Figure 4, the cats in the study were primarily treated with prednisolone at an immunosuppressive dosage (2 mg/kg/day) along with supportive treatment including fluid therapy and supplements. Half of the cases received antibiotics, such as doxycycline, amoxicillin/clavulanic acid, and metronidazole, as initial treatment during the final diagnosis or until bacterial pleuritis or septic peritonitis was ruled out by bacterial culture. Eleven cats eventually died, with survival times ranging from 10 to 140 days. The mean survival time was 38 days, and the median survival time was 31 days. Thirty-two cats were treated with prednisolone but were lost to follow-up. The mean and median survival times for these cats were 28 and 23 days, respectively. The prognosis was estimated to be poor to grave during the last days of recording. In addition, two of the cats had been ill for 5 to 6 days before visiting KUVTH. These cats received only symptomatic treatment and were subsequently lost to follow-up.

**Table 2.** Number of nucleated cells and protein concentrations in FIP effusion.

Measurement	*N*	Range	Mean	Median
TNCC (cells/µl)	43	384–50,100	9,207	5,230
Protein (gm/dl)	45	1.7–9.4	6.14	6.00
A:G ratio	43	0.22–0.7	0.43	0.40

A:G ratio= albumin to globulin ratio.

## Discussion

The majority of FIP cases present abdominal distension and dyspnea as the primary clinical appearance. In some cats with effusive FIP, neurological disorders, and uveitis may be observed, or the cats may exhibit a mixed form of FIP. Cats aged less than 2 years were the most significantly affected by FIP, according to previous research [3,4,11]. Nineteen FIP cats were aged less than 1 year, and 18 cats were 1–2 years old. Scottish fold and Persian breeds were the most prevalent among the purebred cat groups, consistent with previous observations [12].

Commonly reported blood profiles of FIP include anemia, neutrophilia, lymphopenia, thrombocytopenia, hyperproteinemia, hyperglobulinemia, a low A:G ratio, and other abnormalities depending on organ involvement [4,11]. The study showed that 45.5% of effusive FIP cats had neutrophilia. As previously reported, both naturally occurring and experimentally induced FIP are characterized by lymphopenia [3,4]. One hypothesis suggests that lymphocyte apoptosis or destruction occurs when FIPV antigens are presented, leading to immunosuppression and an altered cell-mediated immune response [4]. Consistent with reports, we found that 75% of cats had lymphopenia at the time of initial diagnosis, although there was no significant difference in lymphocyte count between the initial and terminal stages of FIP. The anemia observed in FIP can be nonregenerative, primarily resulting from chronic inflammation, or secondary immune-mediated hemolytic anemia [4,13]. In up to 65% of cats with FIP, anemia was found, usually with a mild decrease in hematocrit [13]. Our study findings revealed that 73.3% of FIP cats had anemia, with 72.7% (24/33) at mild and 27.3% (9/33) at moderate levels. However, Yin et al. [14] reported that lymphopenia was present in half of the 127 cases with a strong suspicion of FIP, while 40.2% showed a decrease in PCV and only 34.3% had an increased neutrophil count. For serum biochemistry, our results revealed that albumin decreased in 91.1% of the cats, while globulin increased in 60% of the cats. These findings suggest that hyperglobulinemia and hypoalbuminemia may raise suspicion of FIP. Among the serum biochemical abnormalities, the A:G ratio, particularly when it is 0.4–0.6, is a significant disease indicator.

Rivalta’s test is a rapid, simple, and inexpensive test that can be used to differentiate transudates from exudates. It is performed by adding a drop of effusion to a slightly acetic solution (one drop of 98%–100% acetic acid added to 5–8 ml of distilled water) [3,4]. A negative result indicates that FIP is unlikely, which is very helpful in ruling out FIP. Conversely, a positive result raises suspicion of FIP, which has a high negative predictive value and positive predictive value reaching up to 90%, particularly in young cats [3,4]. However, positive results may occur in cases of bacterial infection or lymphoma, which must be confirmed with other tests.

Survival times of cats with effusive FIP have been reported from days to weeks [15]. Tsai et al. [16] reported a mean survival time of 21.3 ± 19.9 days for cats with effusive FIP. Previous FIP treatments aimed to suppress the over-immune reaction, promote quality of life, and expand survival rates. Prednisolone is a corticosteroid commonly used in the treatment of various inflammatory conditions in both humans and animals. In the context of COVID-19, prednisolone is considered for severe cases with excessive inflammation and cytokine storms [17,18]. Conversely, the use of prednisolone in FIP follows a different rationale. Prednisone and/or other immunosuppressive agents have been widely accepted as FIP treatment options for managing the clinical signs associated with FIP, but the disease outcome remains unclear [3,4,17]. According to the results of the reduction of globulin levels, prednisolone seems to be effective in reducing inflammation. However, progressive anemia is observed in cats with FIP, as previously reported [18]. Mild to moderate anemia has been associated with FIP, but before the terminal stage or death, severe anemia developed in up to 70% of cases. Future studies should also investigate the treatment effect of the combination of prednisolone at the early treatment stage and antivirals in comparison to each antiviral treatment alone.

Interferons have been frequently used in cats with FIP, including human interferon-alpha and recombinant feline interferon-omega (FeIFN-ω), which is licensed for use in Europe, Australia, and Asia [19–21]. Although Ritz et al. [20] found no statistically significant difference in survival times between cats treated with FeIFN-ω and those administered a placebo, it is noteworthy that one cat was able to survive for up to 200 days. Previously, the target 3C-like protease inhibitor (GC376) has been used as a therapeutic agent to treat FIP [22,23]. Although the treatment with GC376 resolved the clinical signs and prolonged the survival time of cats with FIP, relapses occur when the treatment is stopped in many cases [22]. Recently, antiviral activities against FIPV, such as GS-441524, Remdesivir (GS-5734), and adenosine and guanine analogs like molnupiravir, have been reported as highly effective candidates for the curative treatment of FIP [23–25]. Other commercial medications, such as antifungal itraconazole (ITZ), have demonstrated *in vitro* anti-FIPV activity [26]. A combination of ITZ with prednisolone, ITZ with GS-441524, and ITZ with an anti-human TNF-alpha monoclonal antibody have been reported [27,28]. Doxycycline, a tetracycline-class antibiotic that includes other activities such as anti-inflammatory, anti-apoptotic, antivirus effects, and matrix metalloproteinase inhibition, has been used to treat cats affected by FIP [29,30]. Dunowska and Ghosh [30] reported that doxycycline has some inhibitory effects on FIPV replication *in vitro*. However, further studies should be conducted *in vivo* to evaluate its efficacy on FIP cats [30].

Importantly, FIP primarily affects cats and is not known to infect humans, posing no significant risk to human health. Conversely, the novel coronavirus SARS-CoV-2 can infect cats, although transmission from humans to cats is relatively rare, and most infected cats tend to exhibit mild symptoms or are asymptomatic. While FIP is a cat-specific disease, COVID-19 underscores the potential for cross-species transmission, which primarily occurs from humans to animals [31]. As a veterinarian, it is necessary to monitor and track diseases that pass from animals to humans or from humans to animals.

## Conclusion

Clinical signs and laboratory abnormalities of strongly suspected FIP cats revealed the accumulation of high protein with low to moderate inflammatory cell fluid in body cavities, along with mild anemia, lymphopenia, thrombocytopenia, hypoalbuminemia, hyperglobulinemia, and a low A:G ratio. The survival time of cats with effusive FIP treated with prednisolone ranges from 10 to 140 days, with a median survival time of 31 days. The implementation of invasive diagnostic methods proved difficult in clinical practice, and if IHC is unavailable, a specific and rapid antemortem diagnosis for FIP becomes necessary. In addition, it is imperative to explore and discover further effective therapeutic options for FIP that can lead to rapid resolution, fewer adverse effects, and a lower recurrence rate. In summary, similar to COVID-19, prednisolone may be considered in the treatment regimen for severe cases with inflammatory conditions.

## List of abbreviations

FCoV, feline coronavirus; FeIFN-ω, feline interferon-omega; FeLV, feline leukemia virus; FIP, feline infectious peritonitis; FIV, feline immunodeficiency virus; IHC, immunohistochemistry; PCR, polymerase chain reaction; TNCC, total nucleated cell count; TNF, tumor necrotic factor.
